# *WUSCHEL-RELATED HOMEOBOX 8/9* is important for proper embryo patterning in the gymnosperm Norway spruce

**DOI:** 10.1093/jxb/eru371

**Published:** 2014-09-09

**Authors:** Tianqing Zhu, Panagiotis N. Moschou, José M. Alvarez, Joel J. Sohlberg, Sara von Arnold

**Affiliations:** Swedish University of Agricultural Sciences, Department of Plant Biology, Uppsala BioCenter, Linnean Center of Plant Biology in Uppsala, PO-Box 7080, SE-75007 Uppsala, Sweden

**Keywords:** Apical–basal, cell cycle, division plane, embryogenesis, polarity, spruce, *WUSCHEL-RELATED HOMEOBOX*.

## Abstract

Proper embryo development is crucial as that is when the primary body axes are established. In *Arabidopsis*, *AtWOX8* and *AtWOX9*, members of the *WUSCHEL-RELATED HOMEOBOX* (*WOX*) gene family, are critical for embryo development. In Norway spruce, *PaWOX8/9,* which is expressed in embryos, is the homologue of *AtWOX8* and *AtWOX9*. In this work, it is shown that the transcript abundance of *PaWOX8/9* is high during early and late embryogeny and that it decreases when the maturation phase starts. To address the function of *PaWOX8/9* during embryo development, RNAi lines were established to down-regulate the transcript level of *PaWOX8/9*, using both constitutive and inducible promoters. Embryos in the *PaWOX8/9* RNAi lines show an aberrant morphology caused by disturbed orientation of the cell division plane at the basal part of the embryonal mass during early and late embryogeny. In addition, the transcript level of several key cell-cycle-regulating genes, for example, *PaE2FAB-LIKE* and *PaCYCLIN B-LIKE*, are affected in the *PaWOX8/9* RNAi lines. Taken together, our results suggest that *PaWOX8/9* may perform an evolutionarily conserved function as a regulator of the establishment of the apical–basal embryo pattern.

## Summary

The *WUSCHEL-RELATED HOMEOBOX 8/9* is important for correct orientation of the cell division plane and for cell fate determination during embryo pattern formation in the gymnosperm Norway spruce.

## Introduction

In seed plants, the embryo displays the basic body polarities and develops along the apical–basal axis to establish two meristems responsible for post-embryonic growth (Ueda and Laux, 2012). The process that establishes this primary body plan is called embryonic pattern formation. It requires highly regulated spatio-temporal cell division to set up the organ plan and, eventually, the overall shape of the embryo (Berleth and Jurgens, 1993; Traas *et al.*, 1995; Laux *et al.*, 1996). The orientation of the cell division planes is critical as it not only decides the positions but also the fate of the daughter cells (Vandenberg *et al.*, 1995). Knowledge about embryonic pattern formation in plants has, to a large extent, been derived from studies of embryo-defective mutants in the angiosperm model species *Arabidopsis* (*Arabidopsis thaliana*) (Capron *et al.*, 2009; Kanei *et al.*, 2012). By contrast, our knowledge about the molecular regulation of embryo development in conifers is limited, partly owing to the lack of embryo-defective mutants identified. However, the process of somatic embryogenesis has proved to be a valuable tool for studying the regulation of embryo development in conifers. Somatic embryos have been used for studying gene expression and functions (Tahir *et al.*, 2006; Larsson *et al*., 2012*a*, *b*).

Early embryogenesis in *Arabidopsis* proceeds through highly regular cell division and gene expression patterns. In *Arabidopsis*, embryo pattern formation starts with an asymmetric division of the zygote which gives rise to a smaller apical cell and a larger basal cell. In conifers, the zygote undergoes several rounds of nuclear duplication which is not followed by cytokinesis and there is no clear asymmetric cell division defining apical and basal cell lineages (von Arnold *et al.*, 2002). The descendants of the basal daughter of the *Arabidopsis* zygote only divide transversely to form one file of suspensor cells and the uppermost cell will form the hypophysis. BY contrast, the conifer suspensor consists of several files of non-dividing cells, originating from the basal part of the embryonal mass. The embryonal mass and the suspensor are separated by a layer of conifer-specific cells called embryonal tube cells. After the globular stage, the *Arabidopsis* embryo attains a bilateral symmetry when the two cotyledons start to differentiate. However, in conifers, multiple cotyledons are formed resulting in the embryo retaining radial symmetry. Despite the differences in patterning during embryo development between gymnosperms and angiosperms, recent results have shown that central parts of the regulatory network are conserved.

*WUSCHEL-RELATED HOMEOBOX* (*WOX*) genes form a large gene family in plants and exert important functions during all stages of plant development. The role of *WOX* genes during plant development has been studied in detail in *Arabidopsis* (Schoof *et al.*, 2000). The *WOX* gene family members, *AtWOX2*, *AtWOX8* (also named *STIMPY-LIKE*, *STPL*), and *AtWOX9* (also named *STIMPY*, *STIP*) regulate early embryonic patterning in *Arabidopsis*. *AtWOX2* is expressed in descendants of the apical daughter cell of the zygote and *AtWOX8* and *AtWOX9* are expressed in the basal daughter cell descendants (Haecker *et al.*, 2004; Breuninger *et al.*, 2008; Ueda *et al.*, 2011). *AtWOX8* and *AtWOX9* share redundant functions but their expression patterns differ to some extent. In the shoot, *AtWOX9* functions upstream of the *WOX* gene *AtWUSCHEL*, which is required for shoot apical meristem maintenance*. AtWOX9* has also been linked to cell cycle regulation, as *wox9* loss-of-function mutants are rescued by sucrose which stimulates entry into the cell cycle (Wu *et al.*, 2005). Furthermore, *wox8wox9* double mutants show aberrant cell division orientation in the embryo proper and suspensor (Breuninger *et al.*, 2008).

Phylogenetic analyses have divided the *WOX* gene family into three major clades (the modern clade, the intermediate clade and the ancient clade) (van der Graaff *et al.*, 2009). Eleven *WOX* genes have previously been cloned in Norway spruce (*Picea abies*) from both mRNA and genomic DNA and their phylogenetic relationship to other known *WOX* genes has been examined (Hedman *et al.*, 2013). It was found that Norway spruce *WOX* genes are represented in all of the three major clades identified. The major diversification within the *WOX* gene family took place before the split between gymnosperms and angiosperms. Furthermore, there has been a recent expansion within the intermediate clade of the Pinaceae family. The Norway spruce *PaWOX8/9* gene, belonging to the intermediate clade, is most similar in sequence to *AtWOX8* and *AtWOX9* and it is preferentially expressed during embryo development (Palovaara *et al.*, 2010; Hedman *et al.*, 2013). *In situ* hybridization studies have confirmed that *PaWOX8/9* is expressed during early embryo development in Norway spruce (Palovaara *et al.*, 2010).

The functional analysis of *PaWOX8/9* during embryo development is presented here. It is shown that *PaWOX8/9* is important for the correct orientation of the cell division plane and cell fate determination during early embryo pattern formation, which suggests that *PaWOX8/9* performs an evolutionarily conserved function as an important regulator of apical–basal embryo pattern establishment.

## Materials and methods

### Plant material

The embryogenic cell line 61:21 of Norway spruce (*Picea abies* L. Karst) has been used in this study. The cultures were treated as described previously (von Arnold and Clapham, 2008). Briefly, proembryogenic masses (PEMs) were maintained on solidified proliferation medium containing the plant growth regulators (PGRs) auxin and cytokinin. To stimulate differentiation of early embryos (EEs), cultures were transferred to pre-maturation medium lacking PGRs for one week. For the development of late embryos (LEs) and mature embryos (MEs), the cultures were transferred to maturation medium supplemented with abscisic acid (ABA). After partial desiccation the embryos were germinated for two months.

### RNA extraction, cDNA synthesis, and quantitative real-time PCR

To study the expression of *PaWOX8/9* (accession number: GU944670) during embryo development, samples from PEMs, EEs, LEs and MEs were collected for RNA extraction. Sampling was performed at midday and samples were frozen in liquid nitrogen and stored at –80 °C after collection.

Total RNA was isolated using the Spectrum Plant Total RNA kit (Sigma-Aldrich, USA) according to the manufacturer’s instructions. For each sample, 1 µg of total RNA was reverse transcribed with the RevertAid H Minus First Strand cDNA Synthesis Kit (Fermentas, Thermo Scientific, Sweden) using an equimolar ratio of random and oligo-dT primers according to the manufacturer’s instructions.

Quantitative real-time PCR (qRT-PCR) was performed on an iCycler iQ PCR Thermal Cycler using the iQ5 Real-Time Detection System and 96-well PCR plates with adhesive seals (Bio-Rad Laboratories, USA). Three reference genes, *CELL DIVISION CONTROL2 (PaCDC2)*, *ELONGATION FACTOR 1 (PaEF1)*, and *PHOSPHOGLUCOMUTASE* (*PaPHOS*) were used (Vestman *et al.*, 2011). Experiments were set up as previously described (Hedman *et al.*, 2013). Two or three biological replicates, each with three technical replicates were performed for each test. The primer sequences are presented in Supplementary Table S1 at *JXB* online. Significance analysis of transcript abundance was determined by *t* test.

### RNA interference vector construction

To study the function of *PaWOX8/9*, the coding sequence (CDS) of *PaWOX8/9* was amplified from a cDNA library of early somatic embryos of Norway spruce (Hedman *et al.*, 2013). The full-length CDS was subcloned into the pJET1.2/blunt cloning vector using the CloneJET^TM^ PCR Cloning Kit (Fermentas, Thermo Scientific, Sweden). To obtain RNA interference (RNAi), overlapping fragments of *PaWOX8/9* were amplified and were fused to form a hairpin structure for *PaWOX8/9* (see Supplementary Fig. S1 at *JXB* online). *Eco*RI and *Bam*HI enzyme digestion sites were added on forward primers as linkers. The hairpin was confirmed by sequencing. Primers are presented in Supplementary Table S2 at *JXB* online.

Hairpin structures were introduced into pENTR™/D-TOPO® (Invitrogen, Carlsbad, CA, USA) and then inserted by *att* site LR recombination into the destination vector pMDC7 [LexA-VP16-ER (XVE) β-estradiol inducible promoter, which is derived from the pER8 vector and contains the estrogen receptor-based transactivator XVE] (Zuo *et al.*, 2000; Brand *et al.*, 2006) or pMDC32 (35S constitutive promoter) (Curtis and Grossniklaus, 2003). As a control, an artificial microRNA (amiR) hairpin structure (Schwab *et al.*, 2006; Weigel *et al.*, 2008), designed to silence *GUS,* was inserted into pMDC7. Vectors were introduced by electroporation into *Agrobacterium tumefaciens* strain GV3101.

### Transgenic cell lines

Embryogenic cultures were transformed by co-cultivation with *A. tumefaciens* according to the protocol by Wenck *et al.* (1999), with modifications. Briefly, after initial co-cultivation in liquid transformation medium for 5h, the cultures were collected on filter paper and placed on solidified proliferation medium. After 48h, the cultures were transferred to proliferation medium containing 400mg l^–1^ timentin (Duchefa, The Netherlands) and 250mg l^–1^ cefotaxime (Sanofi, France). Four days later, cultures were transferred to proliferation medium containing 400mg l^–1^ timentin, 250mg l^–1^ cefotaxime, and 15mg l^–1^ hygromycin B (Duchefa). Thereafter, the cultures were subcultured to fresh culture medium of the same composition every week.

Stable transformants were selected after 4 weeks, for 35S:*WOX8/9i*, XVE-*WOX8/9i*, and XVE-*amiRGUS* transformed control lines (T-control). Genomic DNA was isolated from PEMs from the selected lines by using the DNeasy Plant Mini Kit (Qiagen, Germany), according to the manufacturer’s instructions. Transformants were confirmed by PCR.

Transcript abundance of *PaWOX8/9* in PEMs was analysed by qRT-PCR. In the case of XVE-*WOX8/9i* lines, the cultures were exposed to β-estradiol (10 µM) for 48h before analysis. Based on the qRT-PCR data, the following lines were selected for further studies: XVE-*WOX8/9i.1*, XVE-*WOX8/9i.2*, XVE-*WOX8/9i.3*, 35S:*WOX8/9i.1*, 35S:*WOX8/9i.2*, 35S:*WOX8/9i.4*, and 35S:*WOX8/9i.6*. In addition, two T-control lines XVE-*amiRGUS.3* (T.3-control) and XVE-*amiRGUS.4* (T.4-control) were selected. For activation of the XVE promoter during maturation the maturation medium was supplemented with 10 µM β-estradiol and the cultures were transferred to fresh medium of the same composition every week.

### Morphological analysis

To assess the effect of *PaWOX8/9* on embryo morphology during early embryogeny and at the beginning of late embryogeny, samples were collected from cultures of untransformed control (U-control), XVE-*WOX8/9i.3* (non-induced and induced) and lines 35S:*WOX8/9i.1*, 35S:*WOX8/9i.2*, 35S:*WOX8/9i.4*, 35S:*WOX8/9i.6*. The samples were solidified by mixing with 2ml of 1.2% (w/v) Seaplaque agarose (FMC BioProducts, USA) in 60mm Petri dishes.

To study the morphology of EEs *(n*=26) from U-control and line 35S:*WOX8/9i.4*, embryos were scanned with a Zeiss 780 confocal microscope (Carl Zeiss AG, Germany) using a 488nm Argon laser under the 20× objective (NA=0.8).

The developmental pathway for embryos with normal or cone-shaped morphology at the beginning of late embryogeny was followed for 10 d. Embryos from U-control cultures and lines 35S:*WOX8/9i.2*, 35S:*WOX8/9i.4*, and 35S:*WOX8/9i.6* were sampled after 2 weeks on maturation medium and transferred to fresh maturation medium. Time-lapse tracking analyses were performed with about 50 embryos per line. Phenotypes were scored every second day.

For analysing the effect of *PaWOX8/9* during embryo maturation, the morphology of embryos in the U-control and XVE-WOX8/9i lines was analysed for up to 10 weeks on maturation medium. β-estradiol (10 µM) was added to the maturation medium either from the start of the maturation treatment or after 2 weeks on maturation medium when LEs had developed. The germination rate was estimated after 2 months on germination medium. The data from phenotypic differences of early and late embryos were assessed in the ANOVA Mixed-model (*R Development Core Team 2008*, ver. 2.15.1).

For histological analysis, mature embryos from line XVE-*WOX8/9i.3* as well as from U-control cultures were fixed according to the protocol by Karlgren *et al.* (2009). The embryos were serial-sectioned (8 µM) using a Zeiss HM 355 microtome. Cell size was measured by image analysis with ImageJ (ver. 1.48g) (Schneider *et al.*, 2012).

### Identification and qRT-PCR analyses of cell-cycle-regulating genes

To analyse if *PaWOX8/9* affects the expression of cell-cycle-regulating genes, the transcript abundance of cell-cycle-regulating genes was compared in PEMs from the U-control and from line 35S:*WOX8/9i.4* by qRT-PCR. Cell-cycle-regulating genes were identified by homology search over the Norway spruce genome database (Nystedt *et al.*, 2013) combined with phylogenetic analysis see Supplementary Fig. S3 and the Supplementary Information at *JXB* online). The accession numbers of the selected genes are presented in Supplementary Table S7 at *JXB* online. Ten of these genes, *PaRETINOBLASTOMA-RELATED PROTEIN-LIKE (PaRBRL)*, two *E2F* family genes (*PaE2FABL*), five *CYCLIN-LIKE* (*PaCYCLs*) genes, *PaMAP KINASE 6-LIKE (PaMPK6L)*, and *PaEXTRA SPINDLE POLES* (*PaESP*) were selected for qRT-PCR analysis. Three reference genes, *PaCDC2, PaEF1*, and *PaPHOS*, were used. The primer sequences are presented in Supplementary Tables S1 and S8 at *JXB* online.

## Results

### Transcript abundance of *PaWOX8/9* during the development of somatic embryos

**Fig. 1.**
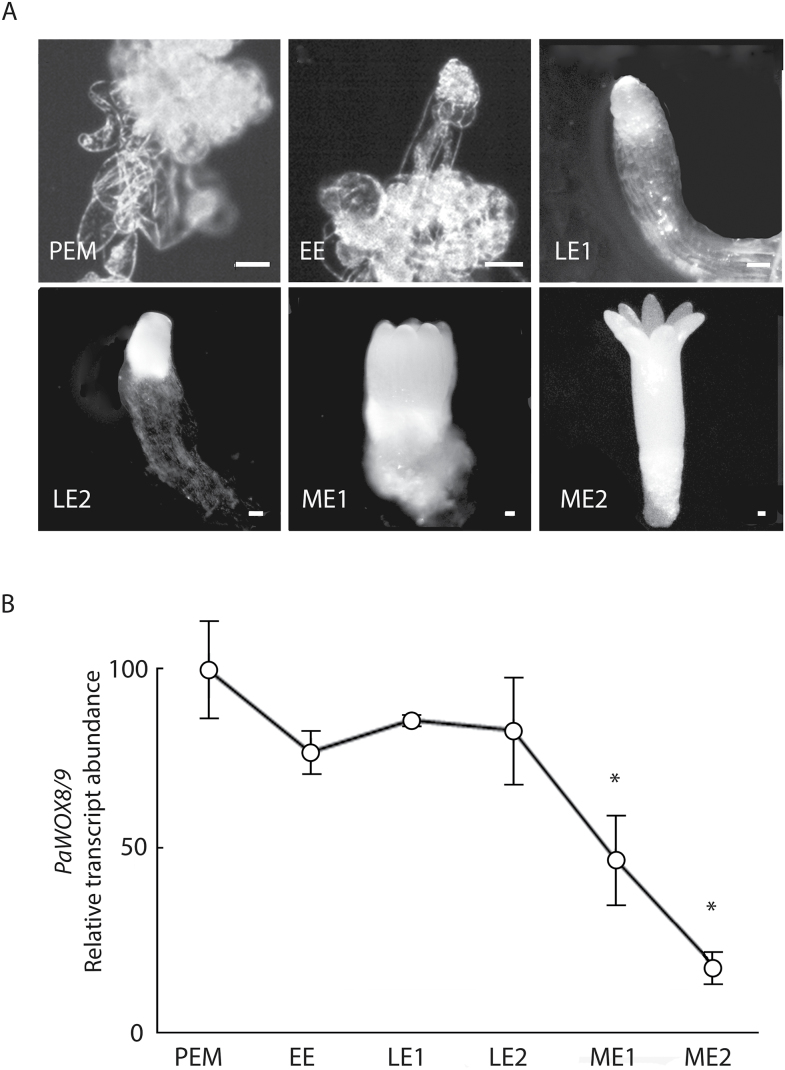
Quantitative real-time PCR analysis of the transcript abundance of *PaWOX8/9* during embryo development. (A) Proliferating proembryogenic mass (PEM) in the presence of the plant growth regulators (PGRs) auxin and cytokinin; early embryo (EE) 1 week after the withdrawal of PGRs, note the protruding embryo from the PEM; early late embryo (LE1) and late embryo before the formation of cotyledons (LE2) after 1 and 2 weeks on the maturation medium, respectively; maturing embryo (ME1) and fully matured embryo (ME2) after 4 and 6 weeks on the maturation medium, respectively. Bar, 100 µm. (B) qRT-PCR analysis of the relative abundance of *PaWOX8/9* in six sequential stages during embryo development, i.e. PEM, EE, LE1, LE2, ME1, and ME2. Transcript levels are relative to the level in PEMs and are normalized against *PaEF1*. The transcript levels are mean ±SE of two biological replicates. Asterisks indicate significant differences in the transcript levels between PEM and later stages (*P* <0.05).

The developmental stages during somatic embryogenesis in Norway spruce have been described (Filonova *et al.*, 2000; Larsson *et al.*, 2012*b*). The stages used in this study include proliferating proembryogenic masses (PEM), early embryos (EE), early late embryos (LE1), late embryos (LE2), maturing embryos (ME1), and fully matured embryos (ME2) (Fig. 1A).

Transcript levels of *PaWOX8/9* were high in PEMs, EEs, LE1s, and LE2s, but significantly lower in ME1s and ME2s (Fig. 1B). *PaWOX8/9* is expressed t the beginning of embryo development in Norway spruce (Palovaara *et al.*, 2010; Hedman *et al.*, 2013). The expression of *PaWOX8/9* in embryos suggests that *PaWOX8/9* is involved in regulating embryo development.

### Transcript abundance of *PaWOX8/9* in RNAi lines

**Fig. 2.**
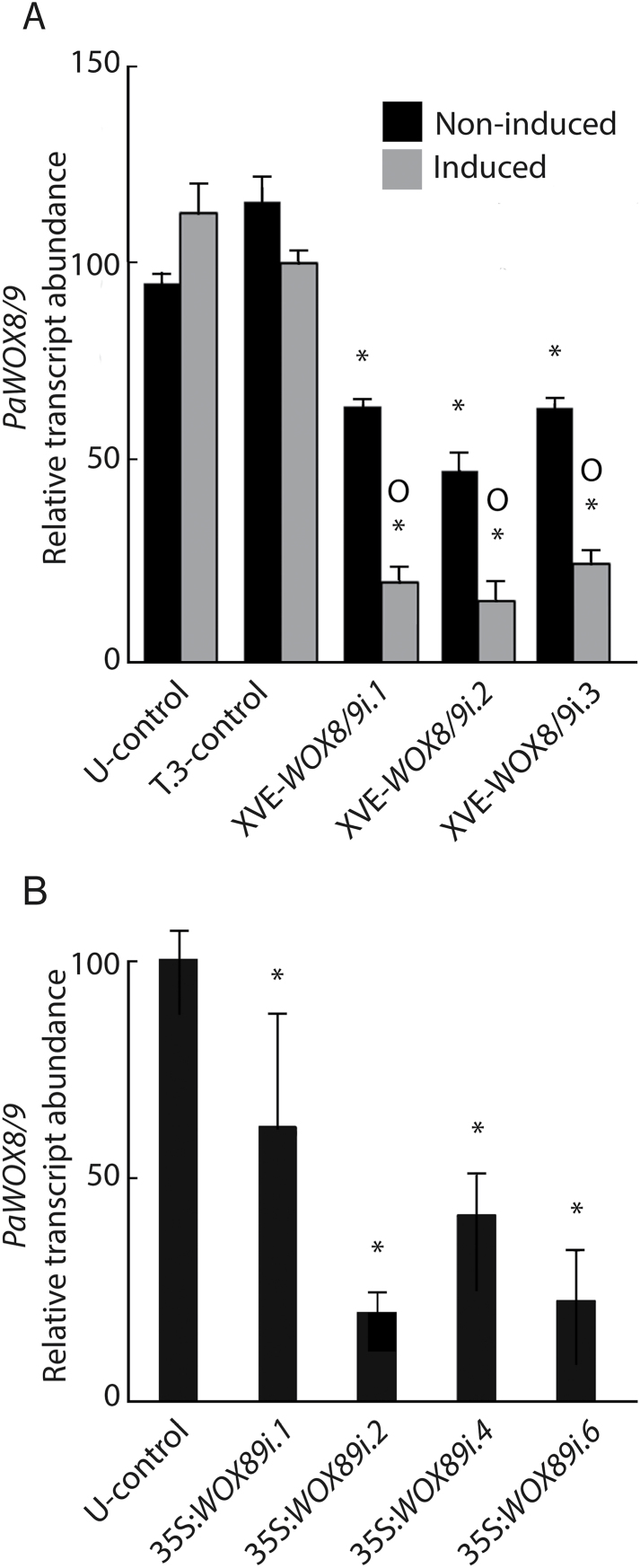
Quantitative real-time PCR analysis of the transcript abundance of *PaWOX8/9* in *PaWOX8/9* RNAi line*s*. Transcript levels are relative to the transcript level in PEMs of the untransformed-control (U-control) and are normalized against three reference genes: *PaCDC2, PaEF1*, and *PaPHOS*. The transcript levels are means ±SE of three biological replicates. (A) qRT-PCR analysis of the relative abundance of *PaWOX8/9* in the U-control, transformed-control line XVE-*amiRGUS.3* (T.3-control), and lines XVE-*WOX8/9i.1*, XVE-*WOX8/9i.2*, and XVE-*WOX8/9i.3,* non-induced (black bars) or induced for 48h with β-estradiol (grey bars). Asterisks indicate significant differences in the transcript levels between the U-control and the XVE-*PaWOX8/9i* lines (*P* <0.05), circles indicate significant differences in the transcript level between the non-induced and induced lines (*P* <0.05). (B) qRT-PCR analysis of the relative abundance of *PaWOX8/9* in the U-control and lines 35S:*WOX8/9i.1,* 35S:*WOX8/9i.2,* 35S:*WOX8/9i.4,* and 35S:*WOX8/9i.6*. Asterisks indicate significant differences in the transcript level between the U-control and the 35S:*PaWOX8/9i* lines (*P* <0.05).

In order to study the function of *PaWOX8/9* during embryogenesis, *PaWOX8/9* RNAi lines were constructed using both inducible and constitutive promoters. Two controls have been used, an untransformed control (U-control) and a transformed control (T-control; described in the Materials and methods). The transcript levels of *PaWOX8/9* in some XVE-*WOX8/9i* lines decreased by more than 50% in PEMs after 48h of β-estradiol treatment (see Supplementary Fig. S2A at *JXB* online). A similar difference in transcript abundance of *PaWOX8/9* in some XVE-*WOX8/9i* lines was also observed after 4 weeks exposure to β-estradiol (data not shown). β-estradiol treatment of the control lines did not significantly affect the transcript abundance of *PaWOX8/9*. Notably, the transcript level of *PaWOX8/9* in non-induced XVE-*WOX8/9i* lines was lower than in the control lines. This implies that the XVE promoter is partly activated in Norway spruce even in the absence of β-estradiol. In most of the 35S:*WOX8/9i* lines, the transcript abundance of *PaWOX8/9* was significantly decreased by *c*. 60% compared with the U-control (see Supplementary Fig. S2B at *JXB* online). Three XVE-*WOX8/9i* lines, four 35S:*WOX8/9i* lines, and one T-control (T.3-control) were selected for further studies (Fig. 2).

### *PaWOX8/9* is required for apical–basal organization in early and late embryos

**Fig. 3.**
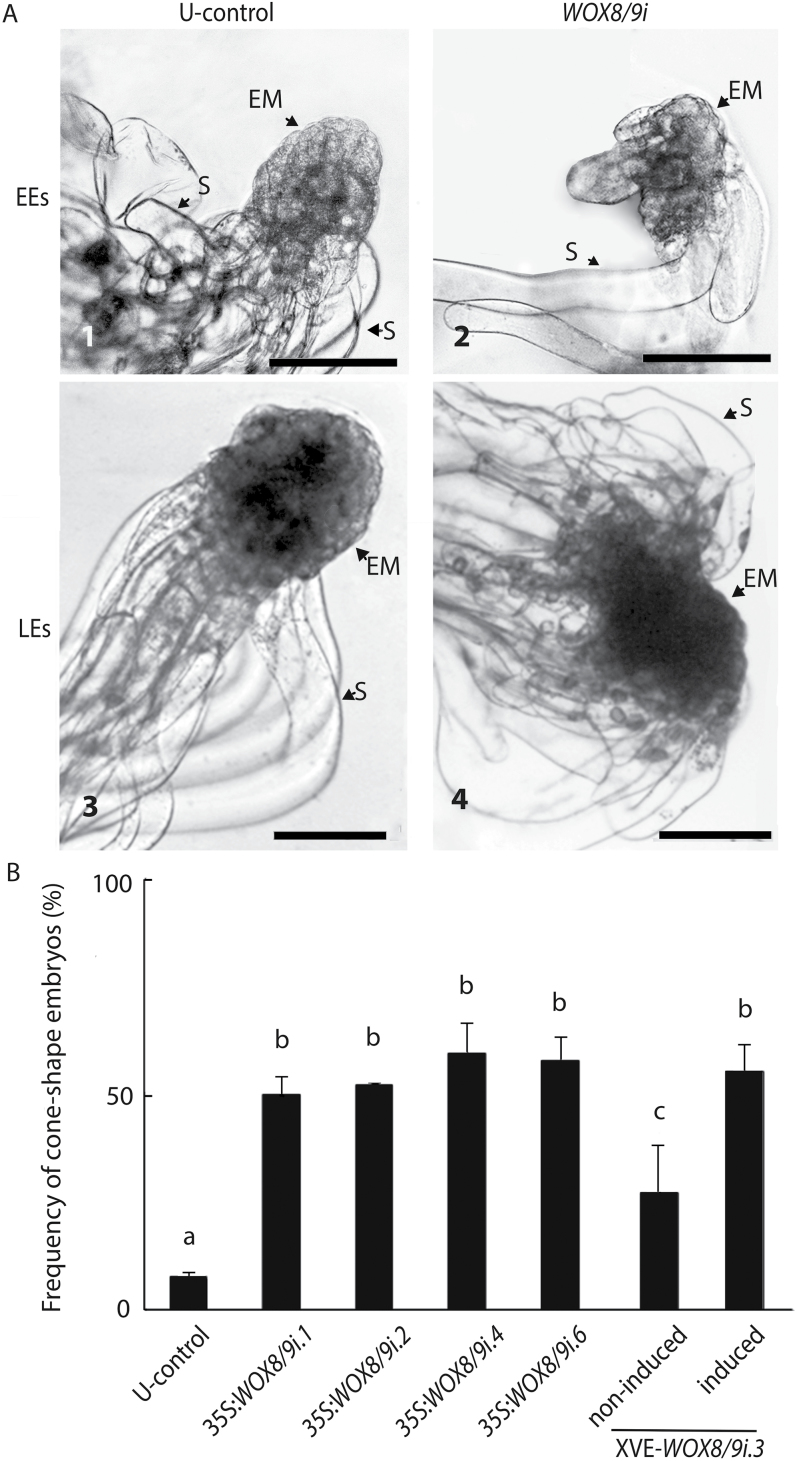
Aberrant morphology of early embryos (EEs) and late embryos (LEs) in *PaWOX8/9* RNAi lines. (A.1) Normal EE from the U-control; (A.2) Aberrant EE from line 35S:*WOX8/9i.4*, note that the embryo lacks a strict border between the embryonal mass and the suspensor; (A.3) Normal LE from the U-control; and (A.4) Cone-shaped LE from line 35S:*WOX8/9i.4,* note the increase in the ratio between the width and length of the embryonal mass. S, suspensor; EM, embryonal mass. Bar, 200 µm. (B) The frequency of cone-shaped LEs in the U-control and lines 35S:*WOX8/9i.1*, 35S:*WOX8/9i.2*, 35S:*WOX8/9i.4*, 35S:*WOX8/9i.6*, and XVE-*WOX8/9i.3* (non-induced and induced). More than 50 LEs were analysed from each line (see Supplementary Table S4 at *JXB* online). Data are presented as means ±SE of three biological replicates. Letters indicate significant differences in the frequency of cone-shaped embryos between the U-control and the transgenic lines (*P* <0.05).

Normally, EEs have a well-delineated rounded embryonal mass in the apical part and vacuolated suspensor cells at the basal part (Fig. 3A.1). More than 95% of the EEs in the control cultures had a normal morphology (see Supplementary Table S3 at *JXB* online). By contrast, more than 50% of the early embryos in all 35S:*WOX8/9i* lines had aberrant morphology. These embryos lacked a strict border between the embryonal mass and the suspensor with suspensor cells differentiating not only from the basal cells in the embryonal mass but also from the upper part (Fig. 3A.2). A high frequency of EEs with aberrant morphology was also observed in the XVE-*WOX8/9i* lines (data not shown). The aberrant morphology resulted in a cone-shaped embryo which was more distinct at the beginning of late embryogeny (Figs 3A.3, 4). LEs with cone-shaped morphology were rarely observed in embryos from control cultures while more than 50% of the embryos in the *WOX8/9i* lines showed a cone-shaped morphology (Fig. 3B; see Supplementary Table S4 at *JXB* online).

**Fig. 4.**
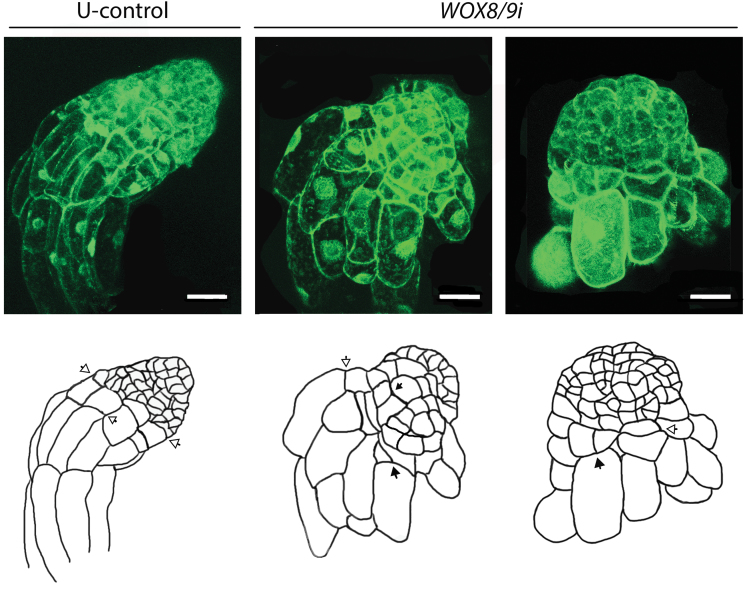
*PaWOX8/9* is important for appropriate orientation of the cell division plane during early embryo development. Images of EEs in the U-control and line 35S:*WOX8/9i.4* were taken using laser scanning confocal microscopy. Images are pseudo-coloured green. Bar, 50 µm. The same embryos are represented schematically below the photographs. Arrowheads show the plane of cell division, (open arrowhead) anticlinal cell division; (solid arrowhead) inclined or periclinal cell division.

To gain further insight into the extent of aberration in embryos from *PaWOX8/9* RNAi lines, embryo morphology was examined using confocal microscopy. Apical–basal polarity is established at the beginning of embryogenesis (Larsson *et al.*, 2008). In control embryos, the cells in the embryonal mass were small with dense cytoplasm. The basally situated cells in the embryonal mass divided anticlinally and the apical daughter cells remained small and non-vacuolated while the basal daughter cells became vacuolated (Fig. 4). In aberrant EEs, in which the transcript level of *PaWOX8/9* was down-regulated, some cells in the embryonal mass were also vacuolated, and the basal cells in the embryonal mass had divided both anticlinally and periclinally. In addition, inclined cell divisions were observed (Fig. 4). These aberrant cell division planes were only observed in embryos from the *PaWOX8/9* RNAi line. Inclined and periclinal divisions of the basal cells in the embryonal mass resulted in suspensor cells developing radially and the strict apical–basal polarity was lost. The embryos developed a cone-shaped morphology. A higher variation in length and width of the suspensor cells was observed in *PaWOX8/9i* embryos than in U-control embryos.

### *PaWOX8/9* promotes embryo maturation

**Fig. 5.**
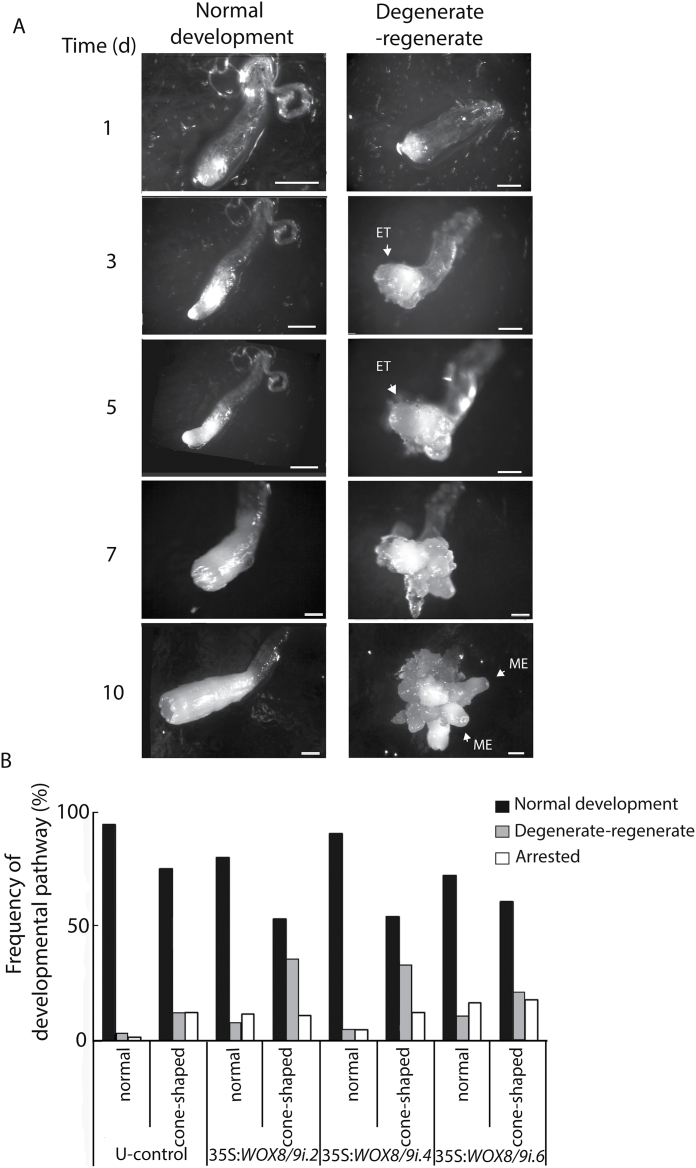
Developmental pathways of normal and cone-shaped embryos. The developmental pathway for embryos with normal or cone-shaped morphology at the beginning of late embryogeny was followed for 10 d. After 2 weeks on maturation medium, embryos from the U-control cultures and lines 35S:*WOX8/9i.2*, 35S:*WOX8/9i.4*, and 35S:*WOX8/9i.6* were isolated and transferred to fresh maturation medium. Time-lapse tracking analyses were performed with about 50 isolated embryos per line. Photographs are presented for day 1, day 3, day 5, day 7, and day 10. (A) The normal and cone-shaped embryos followed two developmental pathways to maturation: (i) normal development; (ii) degeneration–regeneration development, in which embryogenic tissue (open arrowhead, ET) differentiated from the first selected embryo (days 1–5) followed by the development of new MEs (days 7–10, open arrowhead). Bar, 100 µm. (B) Frequency of normal and cone-shaped embryos that developed normally (black bars), went through the degeneration–regeneration pathway (grey bars), or were arrested before maturation (white bars). The presented frequencies are based on the development of 50–90 embryos per line (see Supplementary Table S5 at *JXB* online).

In order to compare the development of normal and cone-shaped embryos, time-lapse tracking analyses were performed. Individual embryos, both normal and cone-shaped, were selected from U-control and *PaWOX8/9* RNAi lines. The embryos were scored every second day. Three developmental pathways were observed: (i) normal embryo maturation (Fig. 5A, Normal); (ii) embryo degeneration-regeneration, in which embryogenic tissue differentiated from the first selected embryo followed by development of new maturing embryos (Fig. 5A, Degenerate–regenerate), and (iii) development arrest, in which embryo development ceased before maturation. Most of the normal embryos in the U-control developed into normal mature embryos (94%); only 4% followed the degeneration–regeneration pathway, and 2% were arrested (Fig. 5B; see Supplementary Table S5 at *JXB* online). The frequency of embryos going through degeneration–regeneration or becoming arrested was slightly higher for cone-shaped embryos in the U-control. The most striking difference between the *PaWOX8/9i* lines and the U-control was a higher frequency of degeneration–regeneration in the cone-shaped embryos, which implies failure in proper early embryonic patterning.

### Morphological defects of MEs in *PaWOX8/9* RNAi lines

**Fig. 6.**
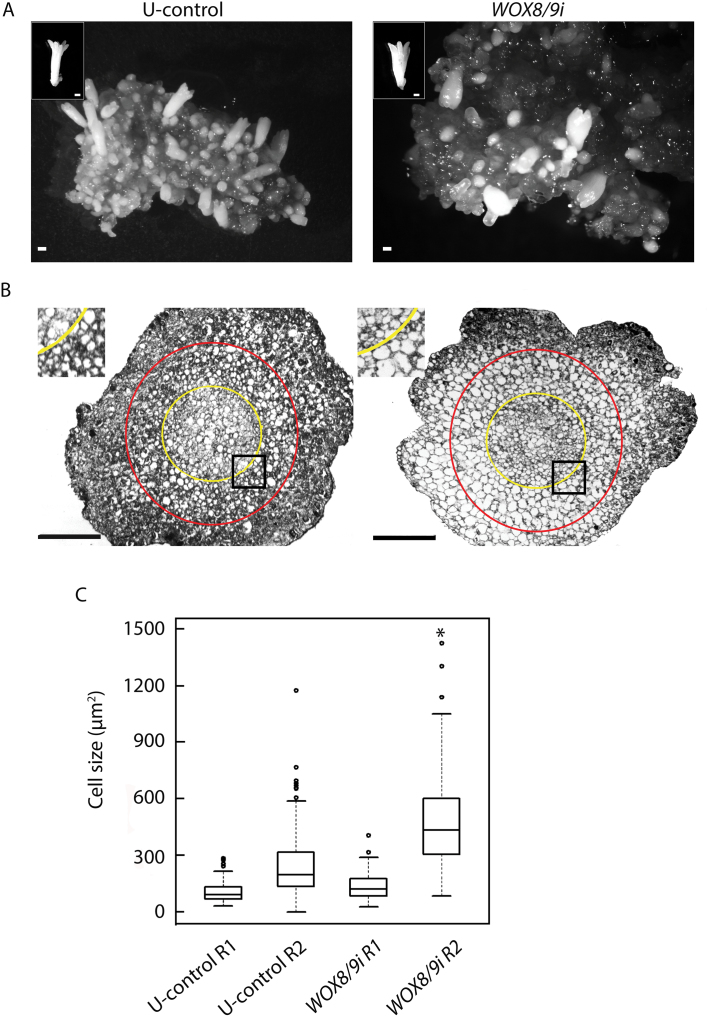
Aberrant morphology of mature embryos (MEs) in *PaWOX8/9* RNAi lines. Embryogenic cultures from the untransformed-control (U-control) and from line XVE-*WOX8/9i.3* were cultured on maturation medium supplemented with β-estradiol for 6 weeks. The cultures were transferred to fresh medium every week. (A) Normal MEs from U-control and heart-shaped MEs from line XVE-*WOX8/9i.3*. Single embryos are presented in the inserts. Bar, 500 µm. (B) Sections of normal and heart-shaped MEs. The area inside the yellow circle, region 1 (R1), includes provascular tissue and pith. The area between the yellow and the red circle, region 2 (R2), includes the inner part of the cortex. The areas in the dark square are presented with higher magnification in the inserts. (C) Boxplot presenting the average cell size of 50 cells for R1 and 100 cells for R2. The data presented are based on sections from four representative embryos. Asterisks indicate significant difference in cell size between the U-control and XVE-*WOX8/9i.3* (*P* <0.001). Bar, 200 µm.

To study the effect of *PaWOX8/9* during maturation, XVE-*WOX8/9i* lines were exposed to β-estradiol either during the whole maturation period or from the third week on maturation medium when LEs had been already developed. On average, 20% of the MEs in control cultures showed a slightly divergent phenotype giving the MEs a more heart-shaped morphology (Fig. 6A). β-estradiol treatment did not affect the frequency of MEs with divergent morphology, nor was the morphology of MEs in line XVE-*WOX8/9i* affected when the β-estradiol treatment started at the third week on maturation medium. However, when the XVE-*WOX8/9i* lines were exposed to β-estradiol during the whole maturation process, a significantly higher frequency of heart-shaped MEs was observed (ranging from 30–50% depending on line). The elongation of the embryos along the apical–basal axis decreased and, instead, the embryos expanded horizontally, resulting in a heart-shaped morphology (Fig. 6A).A similar increase in the frequency of heart-shaped MEs was obtained in all 35S-*WOX8/9i* lines. In sectioned embryos it was obvious that the cortical cells in the heart-shaped embryos were much larger than in normal MEs (Fig. 6B, C). No difference in germination rate was observed between the normal and the heart-shaped embryos.

### *PaWOX8/9* affects the transcript abundance of cell-cycle-regulating genes

**Fig. 7.**
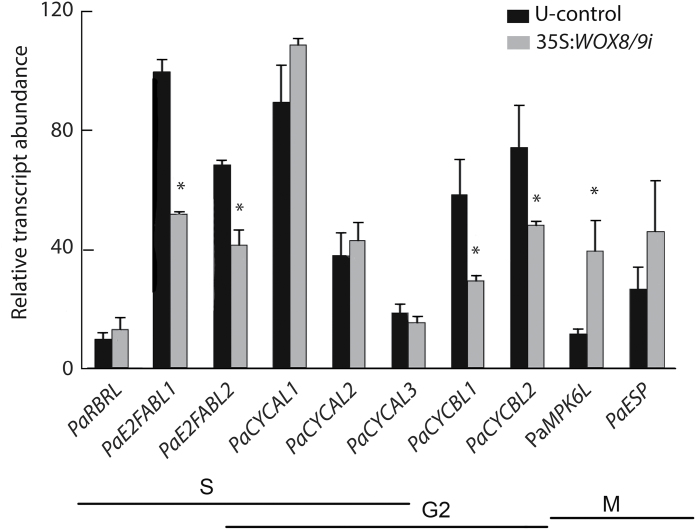
Quantitative real-time PCR analysis of the transcript abundance of cell-cycle-regulating genes in the untransformed control (black bars) and line 35S:*WOX8/9i.4* (grey bars). The transcript level of cell-cycle-regulating genes was analysed in proliferating PEMs in the U-control and in line 35S:*WOX8/9i.4*. The transcript level of the following genes was analysed (for gene accession numbers and primer sequences, see Supplementary Tables S7 and S8 at *JXB* online): *PaRBRL, PaE2FABL1, PaE2FABL2*, and *PaCYCAL1, PaCYCAL2, PaCYCAL3, PaCYCBL1, PaCYCBL2, PaMPK6L*, and *PaESP*. The main functional stages during the cell cycle are illustrated by the lines below. Transcript values are relative to the transcript level of *PaE2FABL1* in PEMs in the U-control and are normalized against three reference genes: *PaCDC2, PaEF1*, and *PaPHOS*. The transcript levels presented are means ±SE of two biological replicates. Asterisks indicate significant differences in transcript level between the U-control and the 35S:*WOX8/9i.4* line (*P* <0.05).

It has been reported that *wox8wox9 Arabidopsis* mutants show aberrant cell division planes (Breuninger *et al.*, 2008). In addition, Weimer *et al.* (2012) reported that asymmetric cell division is controlled by the cell-cycle-regulating gene *AtRBR*. It was reasoned that the observed aberration in the planes of cell division in *PaWOX8/9i* lines implies that *PaWOX8/9* might influence the transcript level of cell-cycle-regulating genes. Therefore, the transcript level of ten cell-cycle-regulating genes (see Supplementary Tables S6 and S7 and the Supplementary Information at *JXB* online) was examined in proliferating PEMs in line 35S:*WOX8/9i.4* and in the U-control. Five out of the ten cell-cycle-regulating genes examined showed significant differences in transcript level between the U-control and the *PaWOX8/9* RNAi line. The transcript abundance of four genes, two *PaE2FABL*s and two *PaCYCBL*s, was significantly decreased in the *PaWOX8/9* RNAi lines (Fig. 7). By contrast, the transcript abundance of *PaMPK6L* was significantly increased (Fig. 7).

## Discussion

In this study it is confirmed that the transcript abundance of *PaWOX8/9* is high during early and late embryogeny and that the abundance decreases when the maturation phase starts and is low in mature embryos (Fig. 1B) (Palovaara *et al.*, 2010; Hedman *et al.*, 2013). To address whether *PaWOX8/9* activity is important for pattern formation during embryo development in Norway spruce, embryogenic RNAi lines were established to down-regulate the transcript level of *PaWOX8/9* using both constitutive and inducible promoters. Embryos in the *PaWOX8/9* RNAi lines showed an aberrant morphology caused by the disturbed orientation of the cell division planes in the basal part of the embryonal mass during early and late embryogeny. However, no difference in embryo development was observed between the control and the XVE-*WOX8/9i* lines when the transcript abundance of *PaWOX8/9* was down-regulated after late embryogeny. Taken together, these results suggest that *PaWOX8/9* plays an important function during differentiation of early embryos in Norway spruce.

In Norway spruce, the apical–basal embryonic pattern formation during early embryogeny proceeds throughout the establishment of the meristematic cells of the embryonal mass and the terminally differentiated vacuolated, expanding suspensor cells (Smertenko *et al.*, 2003). The suspensor cells do not divide but are committed to programmed cell death as soon as they are formed (Filonova *et al.*, 2000; Bozhkov *et al.*, 2005).The existence of stem cells in the basal part of the embryonal mass in Norway spruce is so far conjectural, largely owing to the difficulties of distinguishing these cells under a microscope as they are anatomically more or less similar to the rest of the cells in the embryonal mass. Stem cells continuously produce daughter cells by cell division. Two pools of stem cells can be distinguished: proximal stem cells and distal stem cells (Scheres, 2007). Proximal stem cell daughters form a transitory-amplifying cell population in which extra rounds of cell division take place whereas distal stem cell daughters do not divide again. The only distal stem cell type so far described in plants is the columella stem cells which consist of a single layer of cells below the quiescent centre and their activity generates cells of the central root cap (columella) (Ding and Friml, 2010). It is assumed that basal cells in the embryonal mass in Norway spruce are also distal stem cells which, after asymmetric divisions, give rise to apical meristematic daughter cells in the embryonal mass and basal vacuolated suspensor cells.

Owing to the strictly asymmetric divisions of the stem cells at the basal part of the embryonal mass, early and late embryos in Norway spruce remain polarized. In *PaWOX8/9* RNAi lines the apical–basal polarization is disturbed (Fig. 3A). The early embryos lack a strict border between the embryonal mass and the suspensor and suspensor cells also differentiate from the upper part of the embryonal mass. The aberrant morphology is more distinct in LEs, when the embryonal mass has become cone-shaped (Fig. 3A). By examining EEs in a confocal microscope it was possible to analyse the planes of cell divisions (Fig. 4). In the normal, control embryos, the stem cells at the basal part of the embryonal mass divide anticlinally to give rise to one cell which remains in the embryonal mass and one vacuolated suspensor cell. By contrast, in embryos with decreased transcript levels of *PaWOX8/9*, the basal stem cells of the embryonal mass divide anticlinally, periclinally, and are also inclined. After inclined and periclinal divisions, it appears that both daughter cells remain in the basal part of the embryonal mass. It is likely that the periclinal cell divisions result in radial growth of the embryonal mass, which gives the LEs a cone-shaped morphology. Inclined and periclinal cell divisions might also explain why vacuolated suspensor cells not only develop along the apical–basal axis but also develop radially. It has been shown that stem-cell daughters can adopt different fates depending on their position, and that plant cell fate is spatially determined and requires highly regulated cell division (Scheres *et al.*, 1995; Vandenberg *et al.*, 1995). Our results suggest that (i) the shape of early embryos in Norway spruce depends on the orientation of cell division planes in the stem cells in the basal part of the embryonal mass, and (ii) *PaWOX8/9* directly or indirectly regulates cell division orientation and is important for the cell fate determination required for normal apical–basal organization in early embryos. A similar function has been shown in *Arabidopsis* where *AtWOX8* and *AtWOX9* regulate cell morphology and division pattern in both the basal and the apical lineages from the single-cell embryo stage (Breuninger *et al.*, 2008).

By tracking the development of normal and cone-shaped LEs in control and *PaWOX8/9* RNAi lines, about 30% of the cone-shaped embryos with down-regulated transcript levels of *PaWOX8/9* develop abnormally (Fig. 5). Initially, new embryogenic tissues differentiate from the LEs and, after 1 week, new maturing embryos develop from the embryogenic tissue. It is known that a strict balance between cell division and cell differentiation is essential for normal embryo development and that randomization of cell division planes in embryos leads to drastic morphological defects (Berleth and Jurgens, 1993; Traas *et al.*, 1995; Laux *et al.*, 1996). Although it has not been possible to trace from which cells in the cone-shaped embryos the embryogenic tissue differentiate, it is likely that the division-plane alterations in the stem cells at the basal part of the embryonal mass change cell identity and that some cells have retained meristematic identity.

Decreased transcript abundance of *PaWOX8/9* during the differentiation of EEs and the development of LEs gives a high frequency of cone-shaped LEs (Fig. 3A.4) and heart-shaped MEs (Fig. 6A). However, if the transcript level of *PaWOX8/9* is down-regulated later during the maturation process, the embryos develop into normal cotyledonary embryos. This suggests that *PaWOX8/9* is important during early embryo development but not at late embryogeny. The heart-shaped MEs observed in the *PaWOX8/9* RNAi lines (Fig. 6A) resemble those of the *AtWOX9* mutant *stip-1* in *Arabidopsis* which fail to elongate along the apical–basal axis and cells expand horizontally (Wu *et al.*, 2007).

Cell division is regulated through a complex array of molecular events. In *Arabidopsis*, these events involve factors like sucrose and auxin, which stimulate the expression of *CYCD*. CYCD together with cyclin-dependent kinase (CDK) phosphorylates RBR1 which consequently is released from the E2F/dimerization partner (DP) complex. The E2F/DP complex is then activated to promote (E2FA or E2FB) or inhibit (E2FC) the entrance into the S phase. The RBR/E2F/DP pathway is conserved as both animals and plants use it to control the G1-S transition (Cruz-Ramirez *et al.*, 2012). During the S phase, CYCA forms a complex with CDKA. CYCA is then replaced by CYCB, which stimulates entrance into mitosis (Inzé and De Veylder, 2006; Nieuwland *et al.*, 2009). ESP promotes the metaphase to anaphase transition and cell polarity acquisition (Wu *et al.*, 2010; Moschou *et al.*, 2013). Furthermore, MPK6 is linked to the execution of developmental programmes involving embryogenesis (Hord *et al.*, 2008; Wang *et al.*, 2008).

Our results suggest that *PaWOX8/9* directly or indirectly controls the transcript level of some cell-cycle-regulating genes. The most conspicuous change is the decrease in transcript levels of *PaE2F* and *PaCYCBL* in *PaWOX8/9* RNAi lines (Fig. 7). Interestingly, expression of *AtCYCB*s is under the control of *AtE2F*s (del Pozo *et al.*, 2006). *AtCYCB*s regulate both the G2-M transition and intra-M-phase control (Breyne and Zabeau, 2001; Potuschak and Doerner, 2001).

Over-expression of *AtE2FA* or *AtE2FB* activates mitosis, resulting in more cell proliferation with more cells but of smaller size (Magyar *et al.*, 2012). By contrast, in multi-cellular organisms, a decrease in cell number triggers an increase in mature cell size to compensate for reduced organ size (Ferjani *et al.*, 2007). In accordance, the decrease in the transcript abundance of *PaE2F*s in *PaWOX8/9* RNAi lines might have caused a decrease in cell number by repressing mitosis. The frequency of heart-shaped MEs is higher in *PaWOX8/9* RNAi lines, and in these embryos, the cortical cells are larger than those in normal-shaped MEs (Fig. 6B). It is assumed that the increased frequency of heart-shaped MEs in *PaWOX8/9* RNAi lines is caused by a disturbed cell division pattern during early embryo development.

In conclusion, our results suggest that *PaWOX8/9* acts as an important regulator for establishing the apical–basal embryo pattern and that this function is evolutionarily conserved between gymnosperms and angiosperms. This function is accomplished by controlling the orientation of the cell division planes and cell fate determination during early embryonic pattern formation. In addition, *PaWOX8/9* is active upstream of several important cell-cycle regulators. Future studies will reveal the precise function of *PaWOX8/9* in the regulation of the complex gene regulatory network that determines the symmetry of cell division and how this information is integrated at the whole organismal level to attain proper shape.

## Supplementary data

Supplementary data can be found at *JXB* online.

Supplementary Table S1. Primer sequences used for qRT-PCR of *PaWOX8/9* and corresponding reference genes.

Supplementary Table S2. Primer sequences used for vector construction of *PaWOX8/9* interference.

Supplementary Table S3. Frequency of aberrant early embryos (EEs) in *PaWOX8/9* RNAi lines.

Supplementary Table S4. Frequency of cone-shaped late embryos (LEs) in *WOX8/9* RNAi lines.

Supplementary Table S5. Tracking of the developmental pathway of normal and cone-shaped late embryos (LEs).

Supplementary Table S6. List of the selected cell-cycle-regulating genes from *Arabidopsis thaliana.*

Supplementary Table S7. List of the selected cell-cycle-regulating genes from Norway spruce.

Supplementary Table S8. Primer sequences used for qRT-PCR of the cell-cycle-regulating genes from Norway spruce.

Supplementary Fig S1. Schematic illustration of the CDS of *PaWOX8/9*.

Supplementary Fig S2. Quantitative real-time PCR analysis of the transcript abundance of *PaWOX8/9* in *PaWOX8/9* RNAi line*s*.

Supplementary Fig. S3. Phylogenetic trees of *PaE2FAB-LIKE* genes (A) and *PaCYCLIN-LIKE* genes (B).

Supplementary Information. Identification and selection of cell-cycle-regulating genes in Norway spruce.

Supplementary Data

## Abbreviations:

EE: early embryo
LE: late embryo
ME: mature embryo
PEM: proembryogenic mass
PGR: plant growth regulator.
