# Supplementary material for: WUSCHEL-RELATED HOMEOBOX 8/9 is important for proper embryo patterning in the gymnosperm Norway spruce
Source: J Exp Bot. 2014 Sep 9;65(22):6543–52. doi: 10.1093/jxb/eru371 (PMC4246185; doi:10.1093/jxb/eru371)
Supplement: Supplementary Data [file supp_65_22_6543__index.html]

 WUSCHEL-RELATED HOMEOBOX 8/9 is important for proper embryo patterning in the gymnosperm Norway spruce — WUSCHEL-RELATED HOMEOBOX 8/9 is important for proper embryo patterning in the gymnosperm Norway spruce — Supplementary Data 

# *WUSCHEL-RELATED HOMEOBOX 8/9* is important for proper embryo patterning in the gymnosperm Norway spruce

## Supplementary Data

Data files

**Files in this Data Supplement:**

- Supplementary Data - Supplementary Data
- Supplementary Data - Supplementary Data
- Supplementary Data - Supplementary Data
