# Supplementary material for: WUSCHEL-RELATED HOMEOBOX 8/9 is important for proper embryo patterning in the gymnosperm Norway spruce
Source: J Exp Bot. 2014 Sep 9;65(22):6543–52. doi: 10.1093/jxb/eru371 (PMC4246185; doi:10.1093/jxb/eru371)
Supplement: Supplementary Data [file supp_eru371_jexbot127043_file003.pdf]

WUSCHEL-RELATED HOMEOBOX 8/9 is important for proper embryo patterning in the gymnosperm Norway spruce

Tianqing Zhu, Panagiotis N. Moschou, José M. Alvarez, Joel J. Sohlberg and Sara von Arnold

Swedish University of Agricultural Sciences, Department of Plant Biology, Uppsala BioCenter, Linnean Center of Plant Biology in Uppsala, PO-Box 7080, SE-75007 Uppsala, Sweden

Supplementary Tables

Table S1. Primer sequences used for qRT-PCR of *PaWOX8/9* and corresponding reference genes.

Gene	Forward primer	Reverse primer
PaWOX8/9	GAACAGAAAGTCCAGAACCAAGCAA	GTCCTCGCCTGTTCCTTCATTATAT
*PaWOX8/9i**	CTCAACGTCACACAATCAATGGC	TCTAAGATCCGGAGCTGTTCTGG
PaEF1	CACCTTGGGAGTGAAGCAAATG	GGGAGTAGTGGCATCCATCTTG
PaCDC2	TCCGACGGGTGCAGAGAA	GCTCCATTC AGCCTGATTCAA
PaPHOS	AATGCAGTTGAAGCCATTCC	CCAGTGCCGAAACTCTCTTC

’*’Used for qPCR on mRNA extracted from RNAi lines

Table S2. Primer sequences used for vector construction of *PaWOX8/9* interference.

The underlined sequences show enzyme digestion sites. Sequence in lowercase designates the TOPO-compatible sequence.

Fragment	Forward primer	Reverse primer
1	caccCTCCATGCTGCAGGCTTTCC	GAATTCGGATCCCAAGCAAAGGCAACGCCA
2	GAATTCGGATCCTTTCGTTACACAACACTGGCG	TTGGCGCGCCCTCCATGCTGCAGGCTTTCC

Table S3. Frequency of aberrant early embryos (EEs) in *PaWOX8/9* RNAi lines.

EEs from untransformed control (U-control) and lines 35S:*WOX8/9i.1*, 35S:*WOX8/9i.2*, 35S:*WOX8/9i.4*, 35S:*WOX8/9i.6* were classified as normal (Fig. 3A.1) or aberrant (Fig. 3A.2). Aberrant EEs lacked a strict border between the embryonal mass and the suspensor.

Line	Total number of embryos	Frequency of aberrant EE (%)
U-control	49	4
35S:*WOX8/9i.1*	54	57
35S:*WOX8/9i.2*	30	67
35S:*WOX8/9i.4*	68	69
35S:*WOX8/9i.6*	79	65

Table S4. Frequency of cone-shaped late embryos (LEs) in *PaWOX8/9* RNAi lines.

LEs from U-control, and lines 35S:*WOX8/9i.1*, 35S:*WOX8/9i.2*, 35S:*WOX8/9i.4*, 35S:*WOX8/9i.6* and XVE-*WOX8/9i.3*(induced and non-induced) were classified as either normal (Fig. 3A.3) or cone-shaped (Fig. 3A.4). Cone-shaped indicates LEs with a high width to length ratio of the embryonal mass. The analysis was performed three times. Data from the three replicates are presented separately.

Line	Replicate	Total number of embryos	Frequency of cone-shaped embryos (%)
U-control	a	102	8
	b	155	9
	c	43	7
35S:*WOX8/9i.1*	a	39	54
	b	96	46
	c	160	51
35S:*WOX8/9i.2*	a	21	52
	b	95	53
	c	131	53
35S:*WOX8/9i.4*	a	124	56
	b	81	68
	c	161	55
35S:*WOX8/9i.6*	a	72	60
	b	112	62
	c	178	52
Induced XVE-*WOX8/9i.3*	a	70	59
	b	120	60
	c	85	48
Non-induced XVE-*WOX8/9i.3*	a	47	25
	b	84	39
	c	91	18

Table S5. Tracking of the developmental pathway of normal and cone-shaped late embryos (LEs).

The development of randomly selected LEs with normal and cone-shaped morphology in U-control, *35S:WOX8/9i.2*, *35S:WOX8/9i.4* and 35S:*WOX8/9i.6* lines were followed during 10 days. The LEs were sampled after 2 weeks on maturation medium and transferred to fresh maturation medium. Embryos were grouped according to three developmental pathways (Fig. 4A): i) normal embryo maturation; ii) embryo degeneration-regeneration, in which embryogenic tissue differentiated from the first selected LE followed by development of new maturing embryos and iii) development arrest, in which embryo development ceased before maturation. Asterisks indicate an independent replicate.

Line	Embryo morphology	Number of LEs	Frequency of developmental pathway (%)		
			Normal development	Degeneration-regeneraion	Arrested
U-control	normal	51	94	4	2
	cone-shaped	30	75	12	13
	*normal	71	100	0	0
	cone-shaped	20	85	10	5
35S:*WOX8/9i.2*	normal	25	80	8	12
	cone-shaped	45	54	35	11
35S:*WOX8/9i.4*	normal	41	90	5	5
	cone-shaped	48	55	33	12
	*normal	38	87	3	10
	cone-shaped	48	50	31	19
35S:*WOX8/9i.6*	normal	18	72	11	17
	cone-shaped	33	61	21	18

Table S6. List of the selected cell cycle regulating genes from *Arabidopsis thaliana.*

Gene name	Abbreviation	Protein accession number
AtRETINOBLASTOMA-RELATED PROTEIN	*AtRBR1*	NP_566417
AtMAP KINASE 6	*AtMPK6*	NP_181907
AtEXTRA SPINDLE POLES	*AtESP*	Q5IBC5
AtCYCLIN-A1-1	*AtCYCA1-1*	NP_175077.1
AtCYCLIN-A2-1	*AtCYCA2-1*	NP_197920.2
AtCYCLIN-A2-2	*AtCYCA2-2*	NP_568248.2
AtCYCLIN-A2-3	*AtCYCA2-3*	NP_173010.1
AtCYCLIN-A2-4	*AtCYCA2-4*	NP_178153.1
AtCYCLIN-A3-1	*AtCYCA3-1*	NP_199122.1
AtCYCLIN-A3-3	*AtCYCA3-3*	NP_175155.1
AtCYCLIN-A3-4	*AtCYCA3-4*	NP_175156.1
AtCYCLIN-B1-1	*AtCYCB1-1*	NP_195465.1
AtCYCLIN-B1-3	*AtCYCB1-3*	NP_187759.2
AtCYCLIN-B2-3	*AtCYCB2-3*	Q9LDM4.2
AtCYCLIN-B2-4	*AtCYCB2-4*	NP_177758.2
AtCYCLIN-C1-1	*AtCYCC1-1*	Q9FJK6.2
AtCYCLIN-C1-2	*AtCYCC1-2*	Q9FLQ7.1
AtCYCLIN-D1-1	*AtCYCLD1-1*	P42751.3
AtCYCLIN-D2-1	*AtCYCLD2-1*	P42752.3
AtCYCLIN-D3-1	*AtCYCD3-1*	P42753.3
AtCYCLIN-D3-2	*AtCYCD3-2*	Q9FGQ7.1
AtCYCLIN-D4-1	*AtCYCD4-1*	Q8LGA1.2
AtCYCLIN-D4-2	*AtCYCD4-2*	Q0WQN9.2
AtCYCLIN-D6-1	*AtCYCD6-1*	NP_192236.1
AtCYCLIN-D7-1	*AtCYCD7-1*	NP_195831.1
AtCYCLIN-H-1	*AtCYCH-1*	NP_198114.2
AtCYCLIN-T1-1	*AtCYCT1-1*	NP_174775.1
AtE2FA	*AtE2FA*	Q9FNY0.1
AtE2FB	*AtE2FB*	Q9FV71.1
AtE2FC	*AtE2FC*	Q9FV70.1
AtE2FD	*AtE2FD*	Q9LFQ9.1
AtE2FE	*AtE2FE*	Q8LSZ4.1
AtE2FF	*AtE2FF*	Q8RWL0.1
AtMINICHROMOSOME MAINTENANCE 3	*AtMCM3*	Q9FL33.1

Table S7. List of the selected cell cycle regulating genes from Norway spruce.

Gene name	Abbreviation	Accession number	Protein identity (similarity)	Arabidopsis protein (Acc. number)
PaRETINOBLASTOMA-RELATED PROTEIN-LIKE	*PaRBRL*	MA_1308g0020	65.62 % (76.04 %)	AtRBR1 (NP_566417)
PaMAP KINASE 6-LIKE	*PaMPK6L*	MA_10437020g0010	76.45 % (88.64 %)	AtMPK6 (NP_181907)
PaEXTRA SPINDLE POLES	*PaESP*	HE793991	44.51 % (55.79 %)	AtESP (Q5IBC5)
PaCYCLIN A-LIKE 1	*PaCYCAL1*	MA_88982g0010	57.86 % (76.79 %)	AtCYCA1-1 (NP_175077)
PaCYCLIN A-LIKE 2	*PaCYCAL2*	MA_28323g0010	56.29 % (74.48 %)	AtCYCA1-1 (NP_175077)
PaCYCLIN A-LIKE 3	*PaCYCAL3*	MA_6619g0010	56.57 % (78.29 %)	AtCYCA1-1 (NP_175077)
PaCYCLIN B-LIKE 1	*PaCYCBL1*	MA_10431608g0020	52.65 % (71.21 %)	AtCYCB1-3 (NP_187759)
PaCYCLIN B-LIKE 2	*PaCYCBL2*	MA_19215g0010	43.13 % (56.39 %)	AtCYCB1-3 (NP_187759)
PaE2FAB-LIKE 1	*PaE2FABL1*	MA_66734g0010	50.39 % (66.23 %)	AtE2FA (Q9FNY0)
			56.01 % (66.94 %)	At E2FB (Q9FV71)
PaE2FAB-LIKE 2	*PaE2FABL2*	MA_13142g0010	61.25 % (73.33 %)	AtE2FA (Q9FNY0)
			66.03 % (78.95 %)	At E2FB (Q9FV71)
PaMINICHROMOSOME MAINTENANCE 3-LIKE 1	*PaMCM3L1*	MA_138102g0010	73.26 % (85.63 %)	AtMCM3 (Q9FL33)
PaMINICHROMOSOME MAINTENANCE 3-LIKE 2	*PaMCM3L2*	MA_163650g0010	68.32 % (79.60 %)	AtMCM3 (Q9FL33)

Table S8. Primer sequences used for qRT-PCR of the cell cycle regulating genes from Norway spruce.

Gene	Forward primer	Reverse primer
PaRBRL	TCCCTCAAAGAACTGAGGCTTC	CCGTGTGGGACTCGCAAAA
PaMPK6L	TCATGTGCATCAGCTCCGTTT	TGCTTCCTCCACTGTGATTCTC
PaESP	TAGTCATTGCAGCGAGTTGG	TGCATGCTCTGAACTTCGTC
PaCYCAL1	GACTGGCTTGTTGAGGTTTCAG	TCATATTGCAAGCGGTTCAAA
PaCYCAL2	AAACACCAGGTTGATGCTCC	TTTGGAGGTACGCCTGGTAAT
PaCYCAL3	CCGTGATACAGAGGTGAAGAGAA	TCAGCATGCAAGTGACACCC
PaCYCBL1	CACTGCAATGATTCTAGCTTCCA	GAGCCTGCAGCTTTGAGGAAC
PaCYCBL2	GGGTATCACTGCAATGCTGTTAG	AGCTGCCTTCAAAAACCTGAC
PaE2FABL1	TGGGGCCAATTGATGTTTATCT	ATCAATATTAGCATCCGATGGA
PaE2FABL2	GCTTGGGAAAGAGAAGAGCTGA	TTTGACGCAGGTGTCTGAGGAC
PaMCM3L1	CATCGTTATCGCTCTTCAGGC	TGCAATATGTTCTGATGCCTCA
PaMCM3L2	TTAACTCGCAGGTTGATTCGG	CACTTTGTAACAATTCCCTCCAC
