# Supplementary material for: WUSCHEL-RELATED HOMEOBOX 8/9 is important for proper embryo patterning in the gymnosperm Norway spruce
Source: J Exp Bot. 2014 Sep 9;65(22):6543–52. doi: 10.1093/jxb/eru371 (PMC4246185; doi:10.1093/jxb/eru371)
Supplement: Supplementary Data [file supp_eru371_jexbot127043_file002.pdf]

WUSCHEL-RELATED HOMEOBOX 8/9 is important for proper embryo patterning in the gymnosperm Norway spruce

Tianqing Zhu, Panagiotis N. Moschou, José M. Alvarez, Joel J. Sohlberg and Sara von Arnold

Swedish University of Agricultural Sciences, Department of Plant Biology, Uppsala BioCenter, Linnean Center of Plant Biology in Uppsala, PO-Box 7080, SE-75007 Uppsala, Sweden

Supplementary information

Information S1. Identification and selection of cell cycle regulating genes in Norway spruce.

Since very few cell cycle regulating genes have been identified in Norway spruce, we used a selection of *Arabidopsis thaliana* cell cycle regulating protein sequences (Table S6, Magyar *et al.*, 2005) to run a blast search of the Norway spruce genome database ConGenIE. 12 genes were selected. The gene list with the protein identities/similarities to corresponding Arabidopsis homologues is presented in Table S7. The corresponding primer list for qRT-PCR is presented in Table S8.

Of the selected 12 genes, *PaESP* has already been annotated (accession number: HE793991.1) and an RBR-LIKE protein (accession number: AFB17948.1) has been annotated in *Pinus sylvestris,* and shares 86.41% identity and 90.76% similarity with MA_1308g0020 in the Norway spruce genome database. The remaining ten homologues were identified by blast search. Two CDS, Ma_66734g0010 and Ma_13142g0010 showed similarity to the six Arabidopsis *E2Fs* (*AtE2FA*, *AtE2FB*, *AtE2FC*, *AtE2FD/DEL2*, *AtE2FE/DEL1*, and *AtE2FF/DEL3*) (Berckmans and De Veylder, 2009; Inze and De Veylder, 2006). These putative homologues contain the E2F/DP family winged-helix DNA-binding domain. Ma_66734g0010 and Ma_13142g0010 are closeer related to *AtE2FA* and *AtE2FB* than to *AtE2FC* (Table S6, Fig. S3).

The Arabidopsis *CYCLIN A* (NP_175077.1) was used for the blast of the *CYC* genes. Twenty CDS that belong to the *CYCLIN* gene family were found in the Norway spruce genome database. Out of these 20, six CDS were excluded from further analyses owning to their low sequence coverage (<300 nt). Another four CDS were omitted since they were annotated as *CYCLIN C* or *N*. Five CDS were randomly selected from the remaining ten CDS: MA_88982g0010, MA_28323g0010, MA_6619g0010, MA_10431608g0020 and MA_19215g0010. MA_88982g0010, MA_28323g0010 and MA_6619g0010 are close to *CYCLIN A*, while MA_10431608g0020 and MA_19215g0010 are close to *CYCLIN B*.

MA_10437020g0010 was identified as the *AtMPK6* homologue. MA_138102g0010 and MA_163650g0010 were identified as *AtMCM3* homologues. However, these genes were not used for analyses since they showed very low expression in PEM (data not shown).

One of the genes which have been used as references in the qRT-PCR analysis *CDC2* is a cell cycle regulating gene. However, no difference was found in the expression level of *CDC2* between the U-control and the *PaWOX8/9* down-regulated line.

References

Berckmans B, De Veylder L. 2009. Transcriptional control of the cell cycle. *Current Opinion in Plant Biology* **12**, 599-605.

Inze D, De Veylder L. 2006. Cell cycle regulation in plant development. *Annu Rev Genet* **40**, 77-105.

Magyar Z, De Veylder L, Atanassova A, Bako L, Inze D, Bogre L. 2005. The role of the Arabidopsis E2FB transcription factor in regulating auxin-dependent cell division. *Plant Cell* **17**, 2527-2541.
