# Supplementary material for: WUSCHEL-RELATED HOMEOBOX 8/9 is important for proper embryo patterning in the gymnosperm Norway spruce
Source: J Exp Bot. 2014 Sep 9;65(22):6543–52. doi: 10.1093/jxb/eru371 (PMC4246185; doi:10.1093/jxb/eru371)
Supplement: Supplementary Data [file supp_eru371_jexbot127043_file001.pdf]

WUSCHEL-RELATED HOMEOBOX 8/9 is important for proper embryo patterning in the gymnosperm Norway spruce

Tianqing Zhu, Panagiotis N. Moschou, José M. Alvarez, Joel J. Sohlberg and Sara von Arnold

Swedish University of Agricultural Sciences, Department of Plant Biology, Uppsala BioCenter, Linnean Center of Plant Biology in Uppsala, PO-Box 7080, SE-75007 Uppsala, Sweden

Supplementary Figures

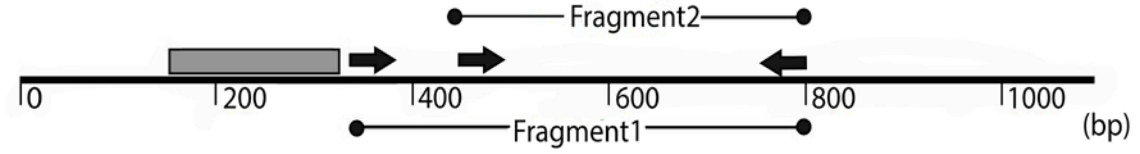

Figure S1. Schematic illustration of the CDS of *PaWOX8/9*. The gray box indicates the homeodomain. The black arrows indicate the location of the primers used for the preparation of the RNAi construct. Primer sequences are presented in table S1.

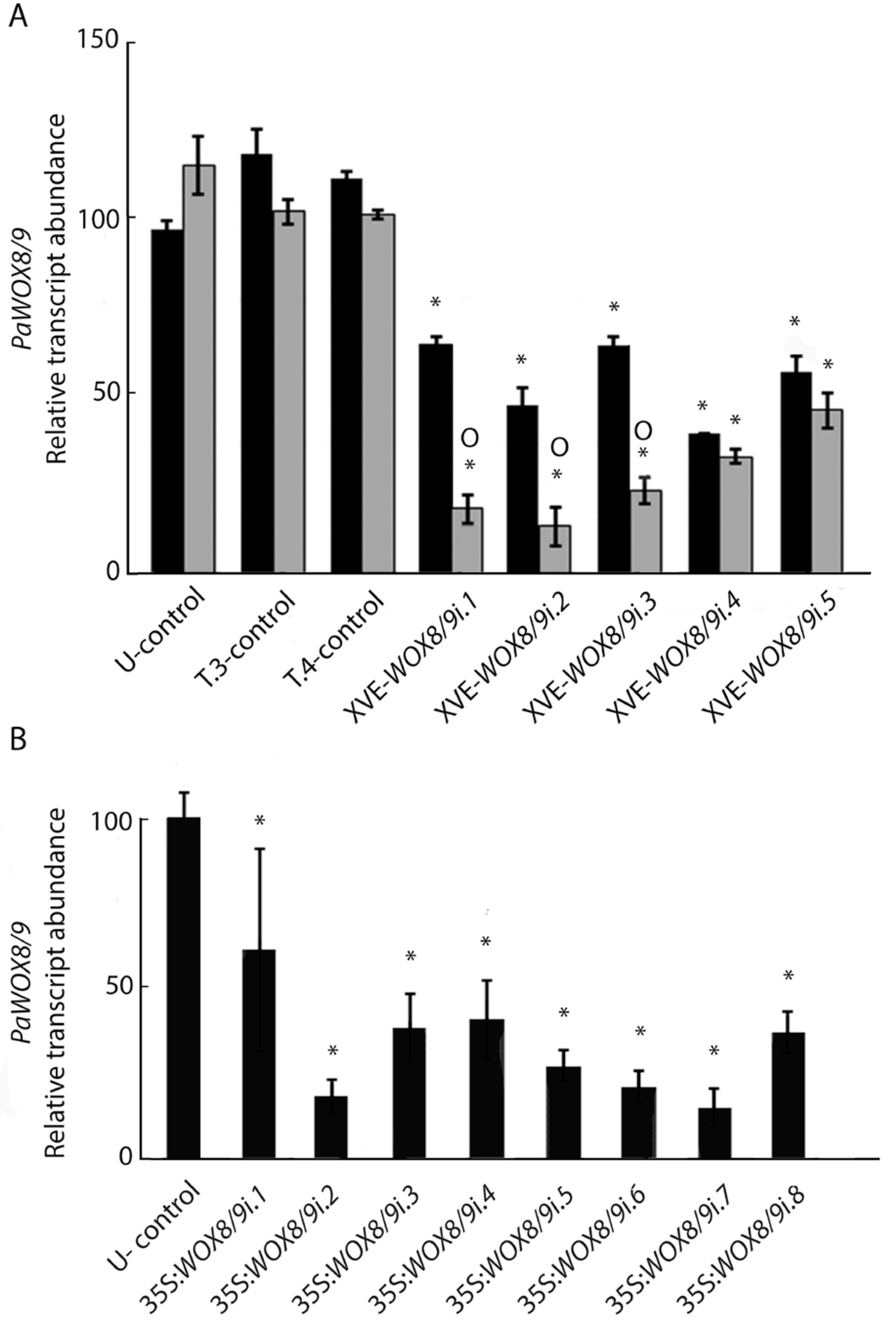
A
150
100
50
0
PaWOX8/9
Relative transcript abundance
U-control
T.3-control
T.4-control
XVE-WOX8/9i.1
XVE-WOX8/9i.2
XVE-WOX8/9i.3
XVE-WOX8/9i.4
XVE-WOX8/9i.5
B
100
50
0
PaWOX8/9
Relative transcript abundance
U- control
35S:WOX8/9i.1
35S:WOX8/9i.2
35S:WOX8/9i.3
35S:WOX8/9i.4
35S:WOX8/9i.5
35S:WOX8/9i.6
35S:WOX8/9i.7
35S:WOX8/9i.8

Figure S2. Quantitative real-time PCR analysis of the transcript abundance of *PaWOX8/9* in *PaWOX8/9* RNAi lines. Transcript levels are relative to the transcript level in PEMs of untransformed control (U-control) and normalized against three reference genes: *CELL DIVISION CONTROL2 (PaCDC2)*, *ELONGATION FACTOR 1 (PaEF1)* and *PHOSPHOGLUCOMUTASE (PaPHOS)*. The transcript levels are mean ± SE of three biological replicates. A) qRT-PCR analysis of the relative abundance of *PaWOX8/9* in U-control, transformed control lines XVE-*amiRGUS.3* (T.3-control), XVE-*amiRGUS.4* (T.4-control) and lines XVE-*WOX8/9i* (1-5), non-induced (black bars) and induced for 48h with β-estradiol (grey bars). Asterisks indicate significant differences in the transcript levels between the U-control and the XVE-*PaWOX8/9i* lines ($p<0.05$), circles indicate significant differences in the transcript level between the non-induced and induced lines ($p<0.05$). B) qRT-PCR analysis of the relative abundance of *PaWOX8/9* in U-control and lines 35S:*WOX8/9i* (1-8). Asterisks indicate significant differences in the transcript levels between the U-control and the 35S:*PaWOX8/9i* lines ($p<0.05$).

A

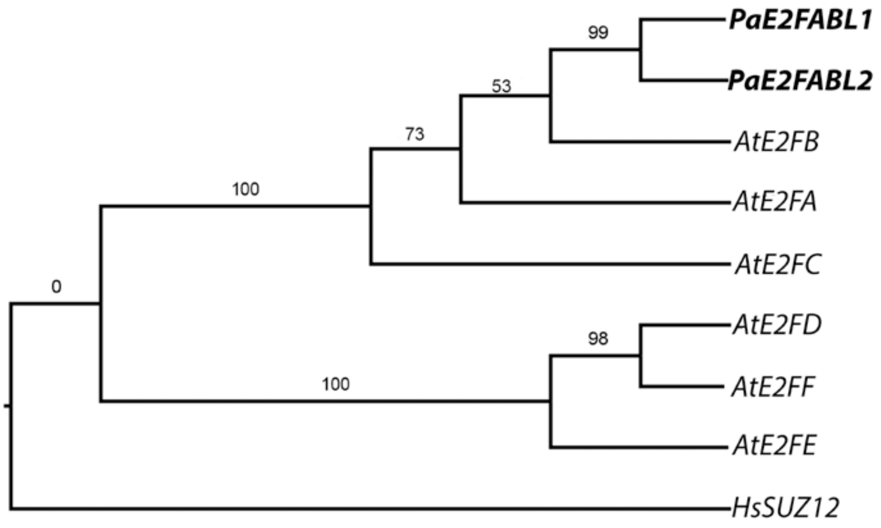
PaE2FABL1
PaE2FABL2
AtE2FB
AtE2FA
AtE2FC
AtE2FD
AtE2FF
AtE2FE
HsSUZ12
99
53
73
100
0
98
100

B

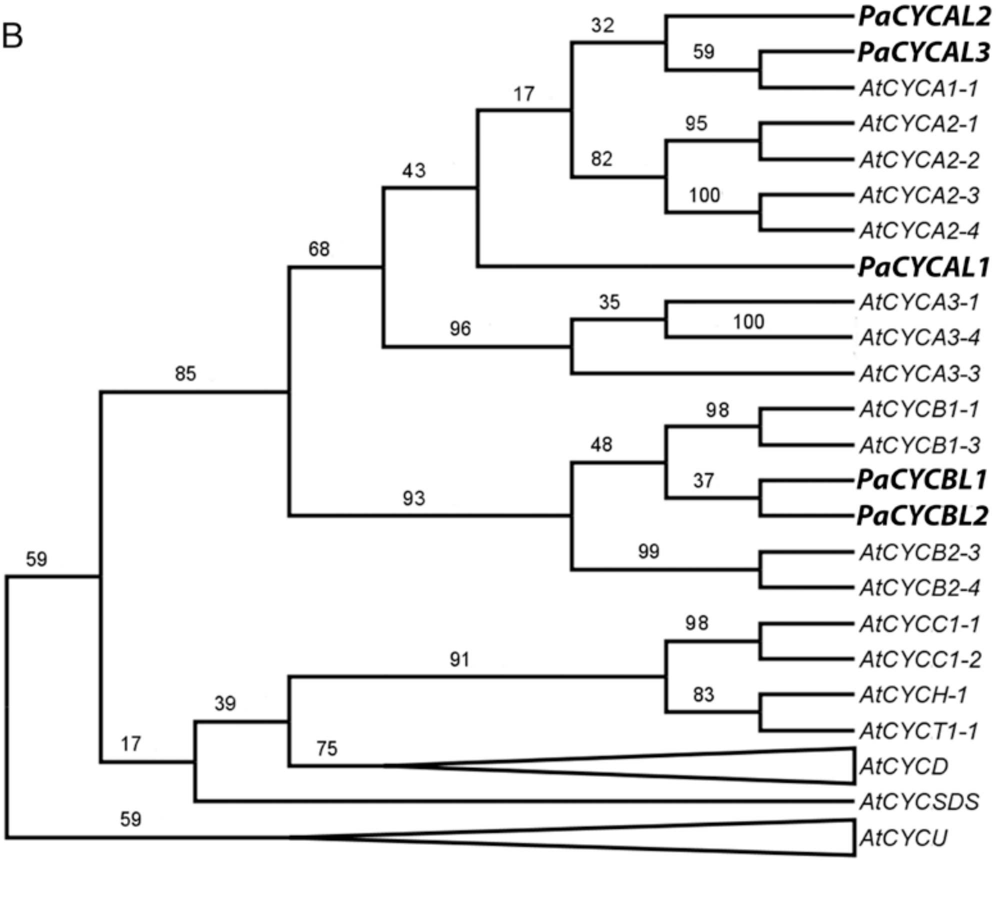
PaCYCAL2
PaCYCAL3
AtCYCA1-1
AtCYCA2-1
AtCYCA2-2
AtCYCA2-3
AtCYCA2-4
PaCYCAL1
AtCYCA3-1
AtCYCA3-4
AtCYCA3-3
AtCYCB1-1
AtCYCB1-3
PaCYCBL1
PaCYCBL2
AtCYCB2-3
AtCYCB2-4
AtCYCC1-1
AtCYCC1-2
AtCYCH-1
AtCYCT1-1
AtCYCD
AtCYCSDS
AtCYCU

Figure S3. Phylogenetic trees of *PaE2FAB-LIKE* genes (A) and *PaCYCLIN-LIKE* genes (B). The trees contain sequences from Arabidopsis (*Arabidopsis thaliana)* and Norway spruce (*Picea abies)*. The Norway spruce sequences are in bold. Norway spruce sequences were translated using Lasergene Editseq (ver 10.0.0 (151, 419)). Accession numbers for Arabidopsis proteins are presented in Table S8. Sequences were aligned using clustalX (ver.2.1) (Larkin *et al.*, 2007). Phylogenetic analyses were performed using maximum likelihood (ML). The ML analyses were performed using Mega (ver.6.0) (Hall, 2013). The JTT + gamma model was used. Support was calculated using the bootstrap method with 500 bootstrap replicates. For the *E2F* tree the human sequence *SUZ12* was used as outgroup, whereas *AtCYC-U* was considered as outgroup for the *CYC* tree. The *AtCYCD* clade is compressed to fit the figure. The resulting trees were edited with fig tree (ver.1.4.0) (http://tree.bio.ed.ac.uk/software/figtree/). Support values indicate bootstrap support using protein data. The tree shown was made using ML and protein data.

Reference

Hall BG. 2013. Building phylogenetic trees from molecular data with MEGA. *Mol Biol Evol* **30**, 1229-1235.

Larkin MA, Blackshields G, Brown NP, Chenna R, McGettigan PA, McWilliam H, Valentin F, Wallace IM, Wilm A, Lopez R, Thompson JD, Gibson TJ, Higgins DG. 2007. Clustal W and Clustal X version 2.0. *Bioinformatics* **23**, 2947-2948.
